# [*N*,*N*-Bis(diphenyl­phosphino)propyl­amine-κ^2^ *P*,*P*′]dichloridoplatinum(II)

**DOI:** 10.1107/S160053680905301X

**Published:** 2009-12-12

**Authors:** Nicoline Cloete, Hendrik G. Visser, Andreas Roodt

**Affiliations:** a Department of Chemistry, University of the Free State, PO Box 339, Bloemfontein 9300, South Africa

## Abstract

The Pt(II) atom in the title compound, [PtCl_2_(C_27_H_27_NP_2_)], has a highly distorted square-planar geometry, as evidenced by the P—Pt—P bite angle [72.4 (1)°]. The strain in the complex is further illustrated by the distorted tetra­hedral angles of the P atoms, which range between 93.5 (1) and 122.2 (1)°. It is of inter­est to note that the N atom has to adopt an almost planar geometry with the two P atoms and the C atom attached to it [it is displaced by 0.093 (2) Å from the CP_2_ plane] in order to accommodate the steric bulk of the phenyl groups and the alkyl group of the ligand coordinated to the Pt^II^ centre. The mol­ecules pack in horizontal rows across the *bc* plane. C—H⋯Cl hydrogen bonds stabilize the crystal packing.

## Related literature

For related platinum(II) complexes, see: Browning *et al.* (1992 ▶); Calabrò *et al.* (2004 ▶); Fei *et al.* (2006 ▶).
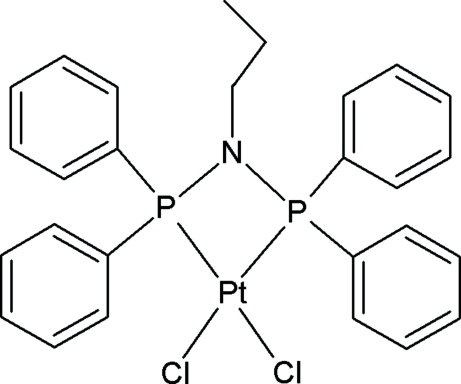


## Experimental

### 

#### Crystal data

[PtCl_2_(C_27_H_27_NP_2_)]
*M* *_r_* = 693.43
Monoclinic, 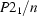

*a* = 10.6301 (4) Å
*b* = 18.8117 (7) Å
*c* = 12.7653 (5) Å
β = 97.326 (1)°
*V* = 2531.84 (17) Å^3^
*Z* = 4
Mo *K*α radiation
μ = 5.90 mm^−1^
*T* = 101 K
0.38 × 0.10 × 0.02 mm

#### Data collection

Bruker X8 APEXII Kappa CCD diffractometer
Absorption correction: multi-scan (*SADABS*; Bruker, 2001 ▶) *T*_min_ = 0.213, *T*_max_ = 0.891
50743 measured reflections
6276 independent reflections
5347 reflections with *I* > 2σ(*I*)
*R*_int_ = 0.052

#### Refinement

*R*[*F* ^2^ > 2σ(*F* ^2^)] = 0.022
*wR*(*F* ^2^) = 0.053
*S* = 1.04
6276 reflections
298 parameters
H-atom parameters constrained
Δρ_max_ = 1.55 e Å^−3^
Δρ_min_ = −0.63 e Å^−3^

### 

Data collection: *APEX2* (Bruker, 2007 ▶); cell refinement: *SAINT-Plus* (Bruker, 2007 ▶); data reduction: *SAINT-Plus*; program(s) used to solve structure: *SIR97* (Altomare *et al.*, 1999 ▶); program(s) used to refine structure: *SHELXL97* (Sheldrick, 2008 ▶); molecular graphics: *DIAMOND* (Brandenburg & Putz, 1999 ▶); software used to prepare material for publication: *WinGX* (Farrugia, 1999 ▶).

## Supplementary Material

Crystal structure: contains datablocks global, I. DOI: 10.1107/S160053680905301X/hy2248sup1.cif

Structure factors: contains datablocks I. DOI: 10.1107/S160053680905301X/hy2248Isup2.hkl

Additional supplementary materials:  crystallographic information; 3D view; checkCIF report

## Figures and Tables

**Table 1:** Selected bond lengths (Å)

Pt—P1	2.1932 (7)
Pt—P2	2.2121 (7)
Pt—Cl1	2.3461 (7)
Pt—Cl2	2.3528 (7)

**Table 2:** Hydrogen-bond geometry (Å, °)

*D*—H⋯*A*	*D*—H	H⋯*A*	*D*⋯*A*	*D*—H⋯*A*
C1—H1*A*⋯Cl1^i^	0.99	2.64	3.512 (3)	147

Symmetry code: (i) 
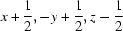
.

**Fig. 1.**
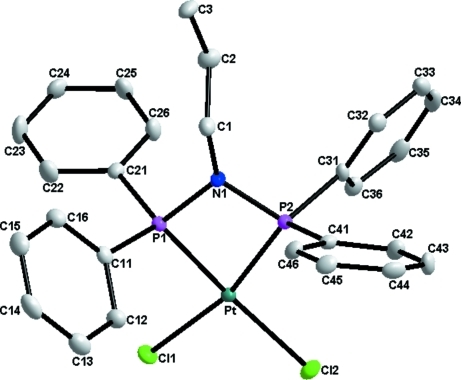
Molecular structure of the title compound. Displacement ellipsoids are drawn at the 50% probability level. H atoms were omitted for clarity.

**Fig. 2.**
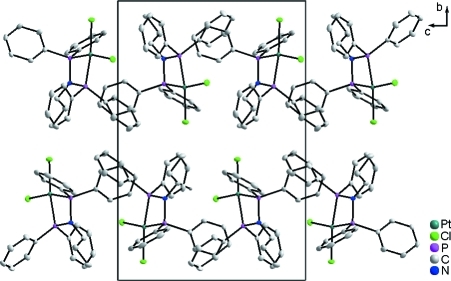
Perspective view of the unit cell of the title compound along the a axis.

## supplementary crystallographic information

### Comment

In the title compound (Fig. 1 and Table 1), all bond distances and angles are considered to be normal and fall within range reported for similar complexes (Browning *et al.*, 1992; Fei *et al.*, 2006; Calabrò *et al.*, 2004). The square-planar geometry of the complex is highly distorted with a P1—Pt—P2 bite angle of 72.40 (3)° and a Cl1—Pt—Cl2 angle of 92.7 (1). The reported P1—Pt—P2 small bite angle forces the P1—N1—P2 angle to 100.1 (1)° which illustrates the distorted geometry from the ideal tetrahedral geometry of the N atom. The distance between the N1 atom and the plane created by C1, P1 and P2 is 0.093 (2) Å. The P atoms are also severely distorted from the expected tetrahedral configuration with Pt—P1—N1 and Pt—P2—N1 angles being 94.0 (1) and 93.5 (1)°, respectively. The molecules of the title compound pack horizontal rows in the unit cell across the *bc* plane (Fig. 2). Intermolecular hydrogen bond exists between C1—H1A and Cl1(1/2+x, 1/2-y, -1/2+z) (Table 2).

### Experimental

Pt(cod)Cl_2_ (50 mg, 0.13 mmol) (cod = 1,5-cyclooctadiene) was dissolved in dichloromethane (15 ml). Bis(diphenylphosphino)propylamine (57.1 mg, 0.13 mmol) was also dissolved in dichloromethane (10 ml) and was added dropwise to the Pt(cod)Cl_2_ solution. The solution was stirred for 2 h at room temperature. The reaction mixture was layered with methanol (10 ml). Colourless single crystals suitable for X-ray crystallography was obtained after 1 d (yield 0.066 g, 73.4%).

### Refinement

H atoms were positioned geometrically and refined as riding atoms, with C—H = 0.95 (aromatic), 0.99 (CH_2_) and 0.98 (CH_3_) Å and with *U*_iso_(H) = 1.2(1.5 for methyl)*U*_eq_(C). The highest residual electron density was found 0.84Å from Pt and the deepest hole 0.68Å from Pt.

### Crystal data

**Table:**

[PtCl_2_(C_27_H_27_NP_2_)]	*F*(000) = 1352
*M**_r_* = 693.43	*D*_x_ = 1.819 Mg m^−^^3^
Monoclinic, *P*2_1_/*n*	Mo *K*α radiation, λ = 0.71073 Å
Hall symbol: -P 2yn	Cell parameters from 6904 reflections
*a* = 10.6301 (4) Å	θ = 2.6–28.2°
*b* = 18.8117 (7) Å	µ = 5.90 mm^−^^1^
*c* = 12.7653 (5) Å	*T* = 101 K
β = 97.326 (1)°	Plate, colourless
*V* = 2531.84 (17) Å^3^	0.38 × 0.10 × 0.02 mm
*Z* = 4	

### Data collection

**Table:**

Bruker X8 APEXII Kappa CCD diffractometer	5347 reflections with *I* > 2σ(*I*)
ω and φ scans	*R*_int_ = 0.052
Absorption correction: multi-scan (*SADABS*; Bruker, 2001)	θ_max_ = 28.3°, θ_min_ = 2.2°
*T*_min_ = 0.213, *T*_max_ = 0.891	*h* = −14→14
50743 measured reflections	*k* = −25→25
6276 independent reflections	*l* = −17→17

### Refinement

**Table:**

Refinement on *F*^2^	0 restraints
Least-squares matrix: full	H-atom parameters constrained
*R*[*F*^2^ > 2σ(*F*^2^)] = 0.022	*w* = 1/[σ^2^(*F*_o_^2^) + (0.0238*P*)^2^ + 1.2293*P*] where *P* = (*F*_o_^2^ + 2*F*_c_^2^)/3
*wR*(*F*^2^) = 0.053	(Δ/σ)_max_ = 0.002
*S* = 1.04	Δρ_max_ = 1.55 e Å^−^^3^
6276 reflections	Δρ_min_ = −0.63 e Å^−^^3^
298 parameters	

### Special details

**Table:**

Experimental. The intensity data was collected on a Bruker X8 Apex II 4 K Kappa CCD diffractometer using an exposure time of 20 s/frame. A total of 1264 frames were collected with a frame width of 0.5° covering up to θ = 28.23° with 99.6% completeness accomplished.

### Fractional atomic coordinates and isotropic or equivalent isotropic displacement parameters (Å^2^)

**Table:**

	*x*	*y*	*z*	*U*_iso_*/*U*_eq_	
N1	0.3830 (2)	0.20629 (12)	0.24699 (19)	0.0122 (5)	
P1	0.27187 (7)	0.19470 (4)	0.33006 (6)	0.01071 (15)	
P2	0.39535 (7)	0.29612 (4)	0.24933 (6)	0.01051 (15)	
Cl1	0.10041 (7)	0.29205 (4)	0.47535 (6)	0.01820 (15)	
Cl2	0.23004 (7)	0.43335 (4)	0.36231 (6)	0.01938 (16)	
Pt	0.250086 (9)	0.308869 (5)	0.357534 (8)	0.00997 (4)	
C1	0.4307 (3)	0.15118 (15)	0.1788 (2)	0.0152 (6)	
H1A	0.4419	0.1731	0.11	0.018*	
H1B	0.3654	0.1136	0.1651	0.018*	
C2	0.5540 (3)	0.11697 (16)	0.2238 (2)	0.0209 (7)	
H2A	0.5478	0.0999	0.2963	0.025*	
H2B	0.6232	0.1525	0.2276	0.025*	
C3	0.5846 (3)	0.05447 (15)	0.1546 (2)	0.0205 (7)	
H3A	0.6649	0.0327	0.1847	0.031*	
H3B	0.5918	0.0715	0.0831	0.031*	
H3C	0.5165	0.0191	0.1518	0.031*	
C11	0.1349 (3)	0.15250 (15)	0.2579 (2)	0.0129 (6)	
C12	0.1292 (3)	0.07992 (16)	0.2354 (2)	0.0178 (6)	
H12	0.1963	0.0495	0.2638	0.021*	
C13	0.0254 (3)	0.05216 (17)	0.1714 (2)	0.0208 (7)	
H13	0.0215	0.0026	0.1569	0.025*	
C14	−0.0725 (3)	0.09605 (17)	0.1286 (2)	0.0210 (7)	
H14	−0.1427	0.0767	0.0842	0.025*	
C15	−0.0678 (3)	0.16837 (18)	0.1507 (2)	0.0204 (7)	
H15	−0.1352	0.1985	0.1219	0.025*	
C16	0.0354 (3)	0.19657 (16)	0.2150 (2)	0.0174 (6)	
H16	0.0385	0.2461	0.2299	0.021*	
C21	0.3394 (3)	0.13850 (15)	0.4372 (2)	0.0123 (6)	
C22	0.4622 (3)	0.15416 (16)	0.4831 (2)	0.0189 (6)	
H22	0.5072	0.1921	0.4557	0.023*	
C23	0.5195 (3)	0.11521 (16)	0.5679 (2)	0.0192 (7)	
H23	0.6035	0.1264	0.5981	0.023*	
C24	0.4550 (3)	0.06004 (16)	0.6087 (2)	0.0189 (6)	
H24	0.4945	0.033	0.6667	0.023*	
C25	0.3330 (3)	0.04456 (17)	0.5647 (3)	0.0255 (7)	
H25	0.2885	0.0066	0.5925	0.031*	
C26	0.2738 (3)	0.08392 (16)	0.4798 (2)	0.0206 (7)	
H26	0.1889	0.0735	0.4511	0.025*	
C31	0.5551 (3)	0.32517 (15)	0.2956 (2)	0.0134 (6)	
C32	0.6533 (3)	0.31537 (15)	0.2338 (2)	0.0174 (6)	
H32	0.6376	0.2925	0.1671	0.021*	
C33	0.7734 (3)	0.33934 (17)	0.2709 (3)	0.0226 (7)	
H33	0.8413	0.3316	0.2305	0.027*	
C34	0.7957 (3)	0.37464 (17)	0.3668 (3)	0.0250 (8)	
H34	0.8786	0.3912	0.3914	0.03*	
C35	0.6988 (3)	0.38594 (16)	0.4269 (3)	0.0205 (7)	
H35	0.7146	0.4109	0.4919	0.025*	
C36	0.5776 (3)	0.36064 (15)	0.3920 (2)	0.0157 (6)	
H36	0.5107	0.3675	0.4337	0.019*	
C41	0.3704 (3)	0.32672 (15)	0.1144 (2)	0.0124 (6)	
C42	0.2932 (3)	0.28739 (15)	0.0390 (2)	0.0152 (6)	
H42	0.253	0.2453	0.0593	0.018*	
C43	0.2749 (3)	0.30953 (15)	−0.0655 (2)	0.0158 (6)	
H43	0.2215	0.283	−0.1167	0.019*	
C44	0.3346 (3)	0.37023 (16)	−0.0948 (2)	0.0169 (6)	
H44	0.3245	0.3846	−0.1667	0.02*	
C45	0.4088 (3)	0.41015 (16)	−0.0201 (2)	0.0182 (6)	
H45	0.4477	0.4525	−0.0406	0.022*	
C46	0.4272 (3)	0.38889 (15)	0.0847 (2)	0.0169 (6)	
H46	0.4782	0.4166	0.136	0.02*	

### Atomic displacement parameters (Å^2^)

**Table:**

	*U*^11^	*U*^22^	*U*^33^	*U*^12^	*U*^13^	*U*^23^
N1	0.0146 (12)	0.0097 (12)	0.0134 (12)	0.0010 (9)	0.0058 (10)	0.0007 (9)
P1	0.0109 (3)	0.0113 (4)	0.0104 (3)	0.0002 (3)	0.0030 (3)	0.0000 (3)
P2	0.0100 (3)	0.0112 (4)	0.0104 (4)	0.0005 (3)	0.0014 (3)	0.0010 (3)
Cl1	0.0176 (4)	0.0213 (4)	0.0176 (4)	0.0008 (3)	0.0093 (3)	−0.0008 (3)
Cl2	0.0240 (4)	0.0116 (3)	0.0225 (4)	0.0025 (3)	0.0025 (3)	0.0003 (3)
Pt	0.01023 (6)	0.01022 (6)	0.00961 (6)	0.00052 (4)	0.00187 (4)	−0.00044 (4)
C1	0.0176 (15)	0.0141 (15)	0.0151 (15)	−0.0014 (12)	0.0065 (12)	−0.0006 (11)
C2	0.0232 (17)	0.0208 (17)	0.0182 (16)	0.0031 (13)	0.0009 (13)	0.0009 (13)
C3	0.0251 (17)	0.0124 (15)	0.0257 (17)	0.0050 (13)	0.0099 (14)	0.0021 (13)
C11	0.0131 (14)	0.0176 (15)	0.0084 (14)	−0.0023 (11)	0.0034 (11)	0.0003 (11)
C12	0.0166 (15)	0.0183 (16)	0.0192 (16)	0.0002 (12)	0.0044 (12)	−0.0024 (12)
C13	0.0231 (16)	0.0186 (16)	0.0215 (17)	−0.0059 (13)	0.0056 (13)	−0.0064 (13)
C14	0.0156 (15)	0.0328 (19)	0.0146 (16)	−0.0079 (13)	0.0022 (12)	−0.0028 (13)
C15	0.0168 (15)	0.0304 (18)	0.0140 (15)	−0.0009 (13)	0.0014 (12)	0.0030 (13)
C16	0.0158 (15)	0.0174 (16)	0.0191 (16)	−0.0006 (12)	0.0022 (12)	−0.0012 (12)
C21	0.0148 (14)	0.0111 (14)	0.0117 (14)	0.0015 (11)	0.0039 (11)	−0.0004 (11)
C22	0.0185 (16)	0.0192 (16)	0.0189 (16)	−0.0045 (13)	0.0023 (13)	0.0066 (13)
C23	0.0131 (15)	0.0239 (17)	0.0201 (16)	−0.0035 (12)	−0.0002 (12)	0.0047 (13)
C24	0.0231 (16)	0.0166 (16)	0.0169 (16)	0.0041 (13)	0.0020 (13)	0.0056 (12)
C25	0.0296 (18)	0.0218 (17)	0.0246 (18)	−0.0095 (14)	0.0019 (14)	0.0101 (14)
C26	0.0158 (15)	0.0250 (17)	0.0203 (16)	−0.0040 (13)	−0.0004 (12)	0.0032 (13)
C31	0.0120 (14)	0.0131 (14)	0.0149 (15)	−0.0023 (11)	0.0006 (11)	0.0045 (11)
C32	0.0177 (15)	0.0172 (16)	0.0175 (16)	0.0015 (12)	0.0026 (12)	0.0065 (12)
C33	0.0148 (15)	0.0212 (17)	0.0321 (19)	0.0021 (13)	0.0043 (14)	0.0129 (14)
C34	0.0143 (15)	0.0197 (17)	0.038 (2)	−0.0043 (13)	−0.0084 (14)	0.0147 (14)
C35	0.0249 (17)	0.0144 (15)	0.0191 (16)	−0.0019 (13)	−0.0096 (13)	0.0035 (12)
C36	0.0176 (15)	0.0145 (15)	0.0140 (15)	0.0002 (12)	−0.0016 (12)	0.0043 (11)
C41	0.0129 (14)	0.0129 (14)	0.0114 (14)	0.0033 (11)	0.0020 (11)	0.0011 (11)
C42	0.0176 (15)	0.0114 (14)	0.0160 (15)	0.0007 (11)	0.0003 (12)	−0.0003 (11)
C43	0.0207 (16)	0.0157 (15)	0.0106 (14)	0.0026 (12)	0.0001 (12)	−0.0038 (11)
C44	0.0183 (15)	0.0221 (16)	0.0099 (14)	0.0077 (12)	0.0002 (12)	0.0024 (12)
C45	0.0177 (15)	0.0178 (16)	0.0189 (16)	−0.0050 (12)	0.0015 (12)	0.0069 (12)
C46	0.0159 (15)	0.0153 (15)	0.0179 (16)	−0.0017 (12)	−0.0039 (12)	0.0011 (12)

### Geometric parameters (Å, °)

**Table:**

N1—C1	1.484 (3)	C21—C22	1.393 (4)
N1—P2	1.695 (2)	C22—C23	1.383 (4)
N1—P1	1.699 (2)	C22—H22	0.95
P1—C21	1.804 (3)	C23—C24	1.383 (4)
P1—C11	1.804 (3)	C23—H23	0.95
Pt—P1	2.1932 (7)	C24—C25	1.378 (4)
P1—P2	2.6019 (10)	C24—H24	0.95
P2—C41	1.804 (3)	C25—C26	1.394 (4)
P2—C31	1.810 (3)	C25—H25	0.95
Pt—P2	2.2121 (7)	C26—H26	0.95
Pt—Cl1	2.3461 (7)	C31—C36	1.394 (4)
Pt—Cl2	2.3528 (7)	C31—C32	1.399 (4)
C1—C2	1.507 (4)	C32—C33	1.379 (4)
C1—H1A	0.99	C32—H32	0.95
C1—H1B	0.99	C33—C34	1.387 (5)
C2—C3	1.530 (4)	C33—H33	0.95
C2—H2A	0.99	C34—C35	1.377 (5)
C2—H2B	0.99	C34—H34	0.95
C3—H3A	0.98	C35—C36	1.392 (4)
C3—H3B	0.98	C35—H35	0.95
C3—H3C	0.98	C36—H36	0.95
C11—C12	1.395 (4)	C41—C46	1.391 (4)
C11—C16	1.400 (4)	C41—C42	1.394 (4)
C12—C13	1.388 (4)	C42—C43	1.388 (4)
C12—H12	0.95	C42—H42	0.95
C13—C14	1.385 (4)	C43—C44	1.381 (4)
C13—H13	0.95	C43—H43	0.95
C14—C15	1.389 (4)	C44—C45	1.380 (4)
C14—H14	0.95	C44—H44	0.95
C15—C16	1.389 (4)	C45—C46	1.386 (4)
C15—H15	0.95	C45—H45	0.95
C16—H16	0.95	C46—H46	0.95
C21—C26	1.390 (4)		
			
C1—N1—P2	132.48 (19)	C14—C15—H15	120
C1—N1—P1	126.38 (19)	C15—C16—C11	120.4 (3)
P2—N1—P1	100.11 (12)	C15—C16—H16	119.8
N1—P1—C21	107.92 (12)	C11—C16—H16	119.8
N1—P1—C11	108.52 (12)	C26—C21—C22	118.8 (3)
C21—P1—C11	110.26 (14)	C26—C21—P1	124.0 (2)
N1—P1—Pt	94.02 (8)	C22—C21—P1	117.1 (2)
C21—P1—Pt	119.51 (10)	C23—C22—C21	120.9 (3)
C11—P1—Pt	114.70 (10)	C23—C22—H22	119.6
C21—P1—P2	123.93 (10)	C21—C22—H22	119.6
C11—P1—P2	122.36 (10)	C22—C23—C24	120.2 (3)
Pt—P1—P2	54.13 (2)	C22—C23—H23	119.9
N1—P2—C41	107.43 (13)	C24—C23—H23	119.9
N1—P2—C31	111.98 (13)	C25—C24—C23	119.4 (3)
C41—P2—C31	103.49 (13)	C25—C24—H24	120.3
N1—P2—Pt	93.46 (8)	C23—C24—H24	120.3
C41—P2—Pt	122.17 (9)	C24—C25—C26	120.9 (3)
C31—P2—Pt	117.65 (10)	C24—C25—H25	119.6
C41—P2—P1	126.30 (10)	C26—C25—H25	119.6
C31—P2—P1	126.28 (10)	C21—C26—C25	119.8 (3)
Pt—P2—P1	53.46 (2)	C21—C26—H26	120.1
P1—Pt—P2	72.40 (3)	C25—C26—H26	120.1
P1—Pt—Cl1	93.70 (3)	C36—C31—C32	120.3 (3)
P2—Pt—Cl1	165.96 (3)	C36—C31—P2	118.8 (2)
P1—Pt—Cl2	172.11 (3)	C32—C31—P2	120.9 (2)
P2—Pt—Cl2	101.35 (3)	C33—C32—C31	119.2 (3)
Cl1—Pt—Cl2	92.67 (3)	C33—C32—H32	120.4
N1—C1—C2	114.9 (2)	C31—C32—H32	120.4
N1—C1—H1A	108.6	C32—C33—C34	120.4 (3)
C2—C1—H1A	108.6	C32—C33—H33	119.8
N1—C1—H1B	108.6	C34—C33—H33	119.8
C2—C1—H1B	108.6	C35—C34—C33	120.7 (3)
H1A—C1—H1B	107.5	C35—C34—H34	119.7
C1—C2—C3	110.3 (3)	C33—C34—H34	119.7
C1—C2—H2A	109.6	C34—C35—C36	119.8 (3)
C3—C2—H2A	109.6	C34—C35—H35	120.1
C1—C2—H2B	109.6	C36—C35—H35	120.1
C3—C2—H2B	109.6	C35—C36—C31	119.6 (3)
H2A—C2—H2B	108.1	C35—C36—H36	120.2
C2—C3—H3A	109.5	C31—C36—H36	120.2
C2—C3—H3B	109.5	C46—C41—C42	119.8 (3)
H3A—C3—H3B	109.5	C46—C41—P2	120.8 (2)
C2—C3—H3C	109.5	C42—C41—P2	119.4 (2)
H3A—C3—H3C	109.5	C43—C42—C41	120.1 (3)
H3B—C3—H3C	109.5	C43—C42—H42	119.9
C12—C11—C16	119.2 (3)	C41—C42—H42	119.9
C12—C11—P1	123.2 (2)	C44—C43—C42	119.7 (3)
C16—C11—P1	117.4 (2)	C44—C43—H43	120.1
C13—C12—C11	120.0 (3)	C42—C43—H43	120.1
C13—C12—H12	120	C45—C44—C43	120.3 (3)
C11—C12—H12	120	C45—C44—H44	119.8
C14—C13—C12	120.6 (3)	C43—C44—H44	119.8
C14—C13—H13	119.7	C44—C45—C46	120.5 (3)
C12—C13—H13	119.7	C44—C45—H45	119.7
C13—C14—C15	119.8 (3)	C46—C45—H45	119.7
C13—C14—H14	120.1	C45—C46—C41	119.5 (3)
C15—C14—H14	120.1	C45—C46—H46	120.2
C16—C15—C14	120.0 (3)	C41—C46—H46	120.2
C16—C15—H15	120		
			
C1—N1—P1—C21	68.6 (3)	C16—C11—C12—C13	−0.3 (4)
P2—N1—P1—C21	−121.87 (13)	P1—C11—C12—C13	−174.2 (2)
C1—N1—P1—C11	−50.9 (3)	C11—C12—C13—C14	0.7 (4)
P2—N1—P1—C11	118.66 (14)	C12—C13—C14—C15	−0.8 (5)
C1—N1—P1—Pt	−168.6 (2)	C13—C14—C15—C16	0.5 (4)
P2—N1—P1—Pt	0.93 (11)	C14—C15—C16—C11	−0.1 (4)
C1—N1—P1—P2	−169.5 (3)	C12—C11—C16—C15	0.0 (4)
C1—N1—P2—C41	42.3 (3)	P1—C11—C16—C15	174.3 (2)
P1—N1—P2—C41	−126.24 (13)	N1—P1—C21—C26	−138.4 (3)
C1—N1—P2—C31	−70.6 (3)	C11—P1—C21—C26	−20.0 (3)
P1—N1—P2—C31	120.79 (14)	Pt—P1—C21—C26	116.1 (2)
C1—N1—P2—Pt	167.6 (2)	P2—P1—C21—C26	−179.4 (2)
P1—N1—P2—Pt	−0.92 (11)	N1—P1—C21—C22	45.4 (3)
C1—N1—P2—P1	168.6 (3)	C11—P1—C21—C22	163.8 (2)
C21—P1—P2—N1	76.89 (17)	Pt—P1—C21—C22	−60.1 (2)
C11—P1—P2—N1	−80.10 (17)	P2—P1—C21—C22	4.4 (3)
Pt—P1—P2—N1	−178.85 (13)	C26—C21—C22—C23	1.5 (5)
N1—P1—P2—C41	72.71 (17)	P1—C21—C22—C23	177.9 (2)
C21—P1—P2—C41	149.61 (16)	C21—C22—C23—C24	−0.3 (5)
C11—P1—P2—C41	−7.38 (17)	C22—C23—C24—C25	−0.4 (5)
Pt—P1—P2—C41	−106.14 (12)	C23—C24—C25—C26	−0.2 (5)
N1—P1—P2—C31	−81.22 (18)	C22—C21—C26—C25	−2.1 (5)
C21—P1—P2—C31	−4.33 (17)	P1—C21—C26—C25	−178.2 (2)
C11—P1—P2—C31	−161.32 (16)	C24—C25—C26—C21	1.5 (5)
Pt—P1—P2—C31	99.93 (12)	N1—P2—C31—C36	−113.0 (2)
N1—P1—P2—Pt	178.85 (13)	C41—P2—C31—C36	131.6 (2)
C21—P1—P2—Pt	−104.26 (11)	Pt—P2—C31—C36	−6.4 (3)
C11—P1—P2—Pt	98.75 (11)	P1—P2—C31—C36	−69.7 (3)
N1—P1—Pt—P2	−0.74 (9)	N1—P2—C31—C32	70.1 (3)
C21—P1—Pt—P2	112.48 (11)	C41—P2—C31—C32	−45.3 (3)
C11—P1—Pt—P2	−113.24 (10)	Pt—P2—C31—C32	176.61 (19)
N1—P1—Pt—Cl1	−178.70 (9)	P1—P2—C31—C32	113.3 (2)
C21—P1—Pt—Cl1	−65.49 (11)	C36—C31—C32—C33	1.9 (4)
C11—P1—Pt—Cl1	68.80 (10)	P2—C31—C32—C33	178.9 (2)
P2—P1—Pt—Cl1	−177.96 (3)	C31—C32—C33—C34	−1.9 (4)
N1—P2—Pt—P1	0.74 (9)	C32—C33—C34—C35	0.4 (5)
C41—P2—Pt—P1	113.85 (12)	C33—C34—C35—C36	1.1 (4)
C31—P2—Pt—P1	−116.32 (11)	C34—C35—C36—C31	−1.1 (4)
N1—P2—Pt—Cl1	9.15 (15)	C32—C31—C36—C35	−0.4 (4)
C41—P2—Pt—Cl1	122.27 (15)	P2—C31—C36—C35	−177.4 (2)
C31—P2—Pt—Cl1	−107.90 (14)	N1—P2—C41—C46	−150.9 (2)
P1—P2—Pt—Cl1	8.41 (11)	C31—P2—C41—C46	−32.3 (3)
N1—P2—Pt—Cl2	−174.28 (9)	Pt—P2—C41—C46	103.3 (2)
C41—P2—Pt—Cl2	−61.16 (12)	P1—P2—C41—C46	169.10 (19)
C31—P2—Pt—Cl2	68.67 (11)	N1—P2—C41—C42	29.1 (3)
P1—P2—Pt—Cl2	−175.02 (3)	C31—P2—C41—C42	147.7 (2)
P2—N1—C1—C2	96.3 (3)	Pt—P2—C41—C42	−76.7 (2)
P1—N1—C1—C2	−97.7 (3)	P1—P2—C41—C42	−10.9 (3)
N1—C1—C2—C3	172.2 (2)	C46—C41—C42—C43	1.1 (4)
N1—P1—C11—C12	78.3 (3)	P2—C41—C42—C43	−178.8 (2)
C21—P1—C11—C12	−39.7 (3)	C41—C42—C43—C44	0.7 (4)
Pt—P1—C11—C12	−178.1 (2)	C42—C43—C44—C45	−2.1 (4)
P2—P1—C11—C12	120.1 (2)	C43—C44—C45—C46	1.7 (5)
N1—P1—C11—C16	−95.8 (2)	C44—C45—C46—C41	0.2 (5)
C21—P1—C11—C16	146.2 (2)	C42—C41—C46—C45	−1.6 (4)
Pt—P1—C11—C16	7.9 (3)	P2—C41—C46—C45	178.4 (2)
P2—P1—C11—C16	−54.0 (3)		

### Hydrogen-bond geometry (Å, °)

**Table:**

*D*—H···*A*	*D*—H	H···*A*	*D*···*A*	*D*—H···*A*
C1—H1A···Cl1^i^	0.99	2.64	3.512 (3)	147

Symmetry codes: (i) *x*+1/2, −*y*+1/2, *z*−1/2.
